# Decline in non-smoking workers’ urine cotinine levels after increased smoking regulation in Korea

**DOI:** 10.1186/s40557-015-0066-z

**Published:** 2015-06-10

**Authors:** Ju-Hyoung Park, Chae-Kwan Lee, Se-Yeong Kim, Chunhui Suh, Kun-Hyung Kim, Jeong-Ho Kim, Byung-Chul Son, Jong-Tae Lee, Seung-Do Yu, Wookhee Choi, Hosub Im

**Affiliations:** Department of Occupational and Environmental Medicine & Institute of Environmental and Occupational Medicine, Pusan Paik Hospital, Inje University, 75, Bokji-ro, Busanjin-gu, Busan 633-165 Republic of Korea; Environmental Health Research Department, National Institute of Environmental Research, Incheon, Republic of Korea, Hwangyong-ro 42, Seogu, Incheon 404-708 Republic of Korea; Center for life & environmental science, Seegene medical foundation, Seoul, Republic of Korea, 320, Cheonho-daero, Seongdong-gu, Seoul 133-847 Republic of Korea

**Keywords:** Urine cotinine, Occupation, Secondhand smoke, Non-smoker

## Abstract

**Objectives:**

To identify any association between implementing smoking regulation policies and workers’ urine cotinine concentration levels in Korea.

**Methods:**

From the first stage of the Korean National Environmental Health Survey conducted by the National Institute of Environmental Research from 2009 to 2011, 2,475 non-smoking workers selected. We analyzed the trend in the changes of cotinine concentration in urine using the general linear model and linear regression, in various jobs as categorized by the National Center for Health Statistics (NCHS) and Korea Standard Classification of Occupations (KSCO).

**Results:**

The urine cotinine concentration tended to decrease every year (2.91 ng/ml in 2009, 2.12 ng/ml in 2010, and 1.31 ng/ml in 2011), showing a decreasing trend (*P* < 0.001). The total subjects’ decreased cotinine concentration in urine between 2009 and 2011 was 2.72 ng/ml (54.1 % relative decrease). The changes in each subgroup’s urine cotinine concentration ranged from 1.59 to 6.03 ng/ml (33.2 to 77.5 %). All groups except for the managerial group (n = 49), which had a small sample size, had statistically significant negative regression coefficients (*p* < 0.05). The ranges of the decrease in urine cotinine were 2.75 ng/ml (53.6 %) for males and 2.72 ng/ml (54.9 %) for females. The negative slope in urine cotinine level was statistically significantly greater in men than women. The changes in urine cotinine by occupation as classified by the NCHS occupational categories ranged from 2.43 to 3.36 ng/ml (46.6 to 61.5 % relative decrease). The negative slopes in urine cotinine levels of the white-collar and farm workers were statistically significantly greater than those of the service workers and blue-collar workers. The change by occupation as classified by the KSCO ranged from 1.59 to 6.03 ng/ml (a 33.2 to 77.5 % relative decrease). The negative slopes in urine cotinine levels of the professionals and related workers and clerks were statistically significantly greater than those of the service workers and plant and machine operators and assemblers.

**Conclusions:**

The cotinine concentration in urine among non-smoking worker groups tended to decline from 2009 to 2011. Such a result may be an indirect indicator of the effectiveness of smoking regulation policies including the revision of the National Health Promotion Act.

## Introduction

Secondhand smoke is defined as the smoke involuntarily inhaled from tobacco smoked by a smoker. It has also been called environmental tobacco smoke (ETS) [1].

Secondhand smoke causes its victims to be exposed to poisonous gases and chemicals such as hydrogen cyanide, carbon monoxide, butane, ammonia, and toluene, as well as toxic metals including arsenic, lead, chromium, and cadmium. Victims are also exposed to more than 50 cancer-causing chemicals such as benzo[*a*] pyrene, tobacco-specific nitrosamines, 4-aminobiphenyl, formaldehyde, benzene, and vinyl chloride. The U.S. Environmental Protection Agency (EPA) and the International Agency for Research on Cancer (IARC) have designated secondhand smoke as a known human carcinogen. The National Institute for Occupational Safety and Health (NIOSH) has also labeled secondhand smoke an occupational carcinogen [2]. Worldwide, 40 % of children, 33 % of non-smoking males, and 35 % of non-smoking females are known to be exposed to ETS [3]. The secondhand smoke exposure rate in Korea (39.7 %) is lower than that of China (49.2 %) but higher than that of Finland (14.3 % in males and 13 % in females) or the U.S. (20.2 %) [4–7]. Notably, in Korea, 58.6 % of male non-smokers and 41.8 % of female non-smokers are exposed to secondhand smoke at worksites [4]. The rates are considerably high, although they are lower than those of Bangladesh (60.8 % in male and 29.6 % in female) or Vietnam (62.8 % in males and 41.3 % in females) [3, 4]. A meta-analysis on studies of the U.S., Europe, China, and Japan in 2007 reported a 24 % increase in the risk for lung cancer among workers exposed to secondhand smoke and a 2.1-fold higher relative risk (95 % CI 1.3-2.6) in a highly exposed group [8]. Furthermore, exposure to secondhand smoke in the workplace is reportedly about four times higher than at home [9], both of which suggest that workers are vulnerable to secondhand smoke.

When a non-smoker is exposed to secondhand smoke, the concentration level of toxic and cancer-causing chemicals in the victim’s body rises more than when a smoker is exposed [10]. Furthermore, even quick exposure to the smoke causes malfunction of the cardiovascular system, increasing the risk for sudden death from a heart attack. The exposure to secondhand smoke at home or in the workplace also increases the risk of lung cancer and heart disease [10].

In Korea, the National Health Promotion Act, first enacted in 1995, went through a number of revisions to strengthen measures for smoking cessation. Through the revision in 2010, local governments were enabled to designate certain places where many people gathered or travelled within their jurisdiction as non-smoking zones. Through the revision in 2011, the range of smoke-free public facilities has been extended.

The purposes of this study include the following: first, to identify the changes in the urine cotinine concentration of non-smoking workers in Korea; second, to indirectly determine the effectiveness of Korean smoking policies, based on the data of the first stage of the Korean National Environmental Health Survey conducted by the National Institute of Environmental Research (NIER) from 2009 to 2011.

## Methods

### Subjects

The raw data used in this study was from the first stage of the Korean National Environmental Health Survey conducted by the NIER from 2009 to 2011, with 6,311 subjects of 3,413 households in 350 enumeration districts nationwide. In selecting regions for the investigation from the raw data, we used districts used in the 2005 Population and Household Census by Statistics Korea as the population. We performed sampling only from the enumeration districts of apartment complexes and regular enumeration districts among the sample enumeration districts of Statistics Korea. Out of the 6,311 subjects of the raw data, we chose 4,853 in the first stage, excluding 40 with missing data and 1,418 who reported that they smoked. Next, we excluded 241 subjects whose urine cotinine concentration was ≥ 50 ng/ml and thus considered smokers [11, 12], and included the remaining 4,612 in the second stage. In this study, cut-off point (50 ng/ml) was determined on the basis of ROC curve’s sensitivity (97.1 %) and specificity (95.1 %). After classifying them into workers and non-workers based on the major categorization of the sixth version of the Korea Standard Classification of Occupations (KSCO), we finally selected 2,475 workers (739 in 2009, 841 in 2010, and 895 in 2011) to include as the subjects of this study.

We collected the subjects’ data on sex, age, residential area, education level, income, smoking status, alcohol consumption status, and residential type from a one-on-one questionnaire in a structured survey form. Those who answered “no” to a question asking whether they were then smoking were classified as non-smokers. Work history was identified from the subjects’ selection of a major category of the sixth version of the KSCO—Those who responded to items number one to nine were regarded as workers, while the rest, except for those in the armed forces, were regarded as non-workers.

### Measures

In the clinical survey, urine samples were collected in a urine specimen cup. After the collection, the samples were transferred in a special icebox manufactured by the researchers, which maintained a temperature of 4-7°. The LOD (limit of detection) of analysis was 0.05 ng/ml. For the analysis of urine cotinine, we extracted cotinine from the urine using the liquid-liquid extraction technique with chloroform. After 1 ml of collected urine was moved to a 10 ml vial, 175 μl of diphenylamine, the internal standard, 50 μl of 0.1 M sodium hydroxide, and 500 μl of chloroform were added. Then the mixture was shaken for one minute. Later, the solution was centrifuged using a centrifugal separator (1,900 g, 10 min), and the supernatant was removed. Finally, 0.1 g of sodium sulfate was added to remove the water and a certain amount was taken to be used as a sample for analysis. For the urine sample analysis, we used a gas chromatography - mass selective detector (Clarus 600 T, PerkinElmer).

### Statistical analysis

We used IBM SPSS Statistics 21.0 for Windows (IBM Corp., Armonk, NY, USA) for data analysis. As the distribution of the subjects’ urine cotinine had a positive skew in the results, we took a natural logarithm of each concentration level and calculated the geometric means and 95 % confidence intervals. To analyze the cotinine concentration in urine of each variable by year, we used a general linear model, and to analyze the slope of the decrease in urine cotinine, we used linear regression. After stratification by year, we compared the urine cotinine concentration levels according to whether the subjects had experienced passive smoking, using an analysis of covariance (ANCOVA), after adjusting for sex, alcohol consumption, residential type. A value of *p* < 0.05 was considered statistically significant.

This study was carried out with the approval of the Institutional Review Board (IRB) (Approval Number: Environmental Epidemiology Division–354) under NIER Rule No. 569 (The NIER IRB Operating Procedure).

## Results

### Determination of cut-off point

In this study, cut-off point of urine cotinine concentration was determined to distinguish smokers from non-smokers. On the basis of ROC curve’s sensitivity (97.1 %) and specificity (95.1 %), the cut-off point of urine cotinine concentration was determined as 50 ng/ml.

### Urine cotinine concentration according to general characteristics

The subjects of this study were 2,475 non-smoking workers surveyed from 2009 to 2011. We analyzed their data by sex, age, residential type, alcohol consumption behavior, and occupation from the major job classifications of the NCHS and KSCO.

Table 1 presents geometric means, and a 95 % CI for each group. The actual decrease in urine cotinine from 2009 to 2011 and the relative changes, coefficients, standard error, and p-value derived from the regression analysis of the logarithm of cotinine concentrations in urine are also presented (Table 1).

**Table 1:** Urine cotinine levels in non-smoking workers, 2009-2011

		2009 (ng/ml)			2010 (ng/ml)			2011 (ng/ml)			Decrease^b^		Slope	SE	p-value
	Total N	N	GM^a^	95%CI	N	GM^a^	95%CI	N	GM^a^	95%CI	2009-2011(%)				
Total	2,475	739	2.91	(2.69-3.14)	841	2.12	(1.95-2.31)	895	1.31	(1.22-1.39)	2.72	(54.1)	−0.403	0.028	<0.001
Sex															
Male	1,132	303	3.10	(2.76-3.47)	398	2.06	(1.83-2.32)	431	1.36	(1.24-1.49)	2.75	(53.6)	−0.412	0.040	<0.001
Female	1,343	436	2.79	(2.51-3.08)	443	2.18	(1.94-2.46)	464	1.26	(1.15-1.38)	2.72	(54.9)	−0.399	0.038	<0.001
Age (yr)															
≤29	199	58	2.52	(1.85-3.43)	67	2.25	(1.69-2.99)	74	1.23	(0.94-1.61)	2.40	(53.1)	−0.368	0.104	<0.001
30-39	420	130	2.87	(2.35-3.50)	141	1.98	(1.64-2.40)	149	1.44	(1.19-1.73)	2.40	(48.6)	−0.345	0.069	<0.001
40-49	639	184	3.11	(2.66-3.63)	203	2.56	(2.21-2.97)	252	1.23	(1.08-1.41)	3.12	(60.4)	−0.476	0.052	<0.001
50-59	689	189	3.10	(2.65-3.62)	231	2.02	(1.75-2.32)	269	1.32	(1.16-1.50)	2.97	(55.0)	−0.426	0.052	<0.001
≥60	528	178	2.70	(2.29-3.17)	199	1.91	(1.64-2.23)	151	1.32	(1.11-1.58)	2.34	(49.3)	−0.355	0.061	<0.001
Residential type															
Detached	972	276	3.12	(2.75-3.54)	331	2.43	(2.17-2.73)	365	1.35	(1.21-1.51)	2.89	(56.7)	−0.428	0.043	<0.001
Tenement	509	129	3.01	(2.45-3.66)	181	2.65	(2.23-3.14)	199	1.59	(1.35-1.87)	2.57	(48.0)	−0.333	0.065	<0.001
Apartment complex	994	334	2.65	(2.35-2.99)	329	1.73	(1.53-1.96)	331	1.15	(1.02-1.30)	2.54	(53.5)	−0.419	0.044	<0.001
Alcohol consumption															
No	1,028	298	2.87	(2.53-3.25)	379	1.93	(1.72-2.16)	351	1.20	(1.06-1.34)	2.92	(59.4)	−0.439	0.044	<0.001
Yes	1,447	441	2.94	(2.62-3.26)	462	2.30	(2.08-2.54)	544	1.38	(1.26-1.52)	2.60	(50.9)	−0.381	0.035	<0.001
NCHS^c^ occup ational categories															
White-collar	789	235	2.61	(2.26-3.00)	271	1.76	(1.51-2.05)	283	1.13	(1.00-1.27)	2.77	(58.3)	−0.419	0.050	<0.001
Service	512	167	3.11	(2.67-3.62)	153	2.38	(1.97-2.87)	192	1.45	(1.25-1.69)	2.43	(46.6)	−0.383	0.057	<0.001
Farm worker	471	147	2.96	(2.44-3.57)	164	2.18	(1.81-2.63)	160	1.18	(1.00-1.39)	3.36	(61.5)	−0.461	0.065	<0.001
Blue-collar	703	190	3.10	(2.07-3.57)	253	2.38	(2.04-2.76)	260	1.50	(1.34-1.69)	2.43	(50.0)	−0.368	0.050	<0.001
KSCO^d^															
Managers	49	10	3.08	(1.20-7.87)	18	1.43	(0.70-2.91)	21	1.31	(0.88-1.95)	6.03	(77.5)	−0.379	0.223	0.095
Professionals and related workers	389	118	2.36	(1.96-2.85)	146	1.77	(1.42-2.21)	125	1.11	(0.92-1.33)	1.82	(45.8)	−0.381	0.075	<0.001
Clerks	351	107	2.86	(2.30-3.55)	107	1.80	(1.43-2.27)	137	1.13	(0.95-1.34)	3.47	(65.0)	−0.466	0.071	<0.001
Service workers	247	88	3.05	(2.44-3.80)	77	2.46	(1.91-3.17)	82	1.75	(1.37-2.24)	2.05	(36.8)	−0.275	0.083	<0.01
Sales workers	265	79	3.19	(2.60-3.91)	76	2.30	(1.73-3.07)	110	1.26	(1.04-1.53)	2.59	(53.6)	−0.469	0.078	<0.001
Skilled agricultural forestry, and fishery workers	471	147	2.96	(2.44-3.57)	164	2.18	(1.81-2.63)	160	1.18	(1.01-1.39)	3.36	(61.5)	−0.461	0.065	<0.001
Craft and related trades workers	215	45	3.43	(2.63-4.46)	83	2.65	(2.03-3.44)	87	1.32	(1.09-1.60)	2.87	(59.7)	−0.507	0.092	<0.001
Plant and machine operators and assemblers	211	47	3.05	(2.27-4.12)	71	2.38	(1.77-3.19)	93	1.87	(1.52-2.30)	1.59	(33.2)	−0.246	0.096	<0.05
Elementary occupations	277	98	2.99	(2.44-3.66)	99	2.17	(1.71-2.76)	80	1.35	(1.11-1.65)	2.85	(57.8)	−0.395	0.079	<0.001

^a^GM, geometric mean

^b^Actual (ng/ml) and relative (%)

^c^NCHS, National Center for Health Statistics

^d^KSCO, Korea Standard Classification of Occupations

The concentration level by year showed a decreasing tendency, from 2.91 ng/ml in 2009 and 2.12 ng/ml in 2010, to 1.31 ng/ml in 2011, and a significant negative regression coefficient (*P* < 0.001). The reduction in the total subjects’ urine cotinine from 2009 to 2011 was 2.72 ng/ml (a 54.1 % relative decrease). The changes in the urine cotinine concentration in each subgroup ranged from 1.59 to 6.03 ng/ml (33.2 % to 77.5 %). All groups except for the managerial group with a small sample size (n = 49) had negative regression coefficients, which were statistically significant. By sex, the range of the decrease in urine cotinine was 2.75 ng/ml for males (53.6 %) and 2.72 ng/ml for females (54.9 %). The negative slope in urine cotinine level was statistically signicantly greater in men than women. By age, the range of the decrease in urine cotinine was between 2.34 and 3.12 ng/ml (a 48.6 to 60.4 % relative decrease). All subgroups had negative regression coefficients, all of which were statistically significant. The range of the decrease in urine cotinine by residential type was between 2.54 and 2.89 ng/ml (a 48 to 56.7 % relative decrease). All subgroups had negative regression coefficients, all of which were statistically significant. By drinking status, the range of the decrease in urine cotinine was 2.92 ng/ml (59.4 %) for non-drinkers and 2.60 ng/ml (50.9 %) for drinkers. The non-drinker group’s negative slope in urine cotinine level was statistically significantly greater than that of the drinking group. The range of the change by the NCHS occupational categories was from 2.43 to 3.36 ng/ml (46.6 to 61.5 % relative decrease). The negative slopes in urine cotinine levels by the NCHS occupational categories were Farm worker (−0.461), White-collar (−0.419), Service (−0.383) and Blue-collar (−0.368) in order, all subgroups were statistically significant. The difference by major category of the KSCO ranged from 1.59 to 6.03 ng/ml (a 33.2 to 77.5 % relative decrease). The negative slopes in urine cotinine levels by major category of the KSCO were craft and related trades workers (−0.507), sales workers (−0.469), clerks (−0.466), skilled agricultural, forestry, and fishery workers (−0.461), elementary occupations (−0.395), professionals and related workers (−0.381), managers (−0.379), service workers (−0.275) and plant and machine operators and assemblers (−0.246) in order, all subgroups except managers were statistically significant.

### Urine cotinine concentration by secondhand smoking status and sex, after stratification by year

In 2009, 2010, and 2011, the cotinine concentration in urine was statistically significantly higher in the group exposed to secondhand smoke than the group without such exposure (*p* < 0.001). The yearly comparison by sex in the non-exposure group showed higher urine cotinine concentration levels in males than females throughout the three years, and the result of 2009 was statistically significant (*p* < 0.05). In the yearly comparison by sex in the secondhand smoke exposure group, the urine cotinine level was found to be higher in women in all three years, and among these, the result of 2010 was statistically significant (*p* < 0.05) (Table 2).

**Table 2:** Urine cotinine concentration by whether the subject was exposed to SHS^a^ by sex

		2009 (ng/ml)					2010 (ng/ml)					2011 (ng/ml)				
		N	GM^†^	95%CI	*p*-value^‡^	*p*-value^§^	N	GM^†^	95%CI	*p*-value^‡^	*p*-value^§^	N	GM^†^	95%CI	*p*-value^‡^	*p*-value^§^
SHS exposure						<0.001					<0.001					<0.001
No	Total	424	2.54	(2.29-2.80)			493	1.75	(1.57-1.94)			521	1.08	(0.99-1.17)		
	Male	171	2.90	(2.48-3.39)	<0.05		226	1.89	(1.56-2.16)	0.428		231	1.17	(1.01-1.31)	0.183	
	Female	253	2.33	(2.05-2.64)			267	1.64	(1.44-1.95)			290	1.01	(0.91-1.15)		
Yes	Total	315	3.49	(3.13-3.94)			348	2.79	(2.47-3.19)			374	1.71	(1.55-1.89)		
	Male	132	3.38	(2.85-4.17)	0.890		172	2.29	(1.96-2.80)	<0.05		200	1.62	(1.40-1.87)	0.285	
	Female	183	3.57	(3.00-4.12)			176	3.40	(2.79-3.97)			174	1.82	(1.56-2.12)		

^a^SHS, second hand smoke

^†^GM, geometric mean adjusted for sex, alcohol consumption, residential type

^‡^Comparison of sex after stratification by whether the subject was exposed to SHS through ANCOVA (adjusted for alcohol consumption, residential type)

^§^Comparison by whether the subject was exposed to SHS through ANCOVA (adjusted for sex, alcohol consumption, residential type)

## Discussion

For evaluation of the secondhand smoke, metabolites such as carbon monoxide, thiocyanate, 4-aminobiphenyl-hemoglobin adduct, benzo[a] pyrene-DNA adduct, PAH-albumin adduct, hydroxyproline, and aromatic amines are known to be available. However, tests based on those substances yield low sensitivity and specificity. Furthermore, they are easily affected by environmental variables [13, 14]. Cotinine is a major metabolite of nicotine and is oxidized by CYP2A6 in the liver. It is spread throughout body fluids including the blood, saliva, and urine [15]. Cotinine is widely used as a metabolite to evaluate the exposure to smoke [16, 17] because of its several advantages: Its half-life (18–24 h) reflects two to three days of cumulative exposure [18]; it is easy to sample and can be sampled non-invasively.; it is not greatly affected by exposure to types of smoke other than cigarette smoke [16, 17]. In this study, we didn’t use the calculation of a cotinine-to-creatinine ratio, because according to the previous study, using the calculation of a cotinine-to-creatinine ratio was not useful exercise in smokers and passively exposed individuals. Uncorrected urine cotinine concentration showed a much stronger correlation with serum concentration than urine cotinine:creatinine ratios [19].

The geometric mean of urine cotinine concentration levels of 2,475 subjects who were non-smoking workers was reported in one study to be lower than the urine concentration levels of 14,315 non-smokers in Korea between 2007 and 2010 [20]. It was also lower than the value reported by another study on 4,084 non-smoking workers in Korea in 2008 [21] and in 629 non-smoking workers in Busan, Ulsan, and Kyeongnam Provinces [22]. When comparing urine cotinine concentrations by secondhand smoking status after stratification by year, the cotinine level was higher in the group who reported that they were exposed to secondhand smoke than in those who said they were not. Such a result is congruent with what was reported in a study conducted in Korea in 2008 [22].

The decrease in urine cotinine concentration was larger in male workers than in female workers, while the decrease rate was higher in the women workers. This finding is in concordance with that of a study on non-smoking workers in the U.S. between 1988 and 2002 [23]. It was also reported in another study that men’s urine cotinine concentration was higher than women’s [24]. In addition, socioeconomic factors have been found to influence the severity of exposure to secondhand smoke [25]. These phenomena are thought to be due to the fact that men are more frequently and intensely exposed to ETS than women because of socioeconomic factors and their higher smoking rates. This could mean that the frequency and intensity of men’s exposure to secondhand smoking would be more influenced by the enforcement of smoking regulations. By age group and residential type, all subgroups showed a decreasing trend in cotinine concentration in urine, with variations in the amount and rate of the decrease. This is thought to be caused by different working environments by age group, lifestyle, and the frequency and severity of exposure to ETS by residential type, as socioeconomic factors such as income and education level affect the degree of the exposure to secondhand smoking [25]. Non-drinkers showed a larger amount and rate of decrease in the urine cotinine concentration than drinkers. Considering a report that bar or restaurant workers had ≥ 10 times higher cotinine concentration in saliva than office workers [26], such a result suggests that drinkers experience more frequent and intense exposure to ETS in restaurants or bars than non-drinkers. Therefore, it is necessary to strengthen smoking regulations at restaurants or bars.

Under the NCHS occupation categories, the rates of decrease in urine cotinine of the white-collar workers and farm workers were higher than those of the service workers and blue-collar workers. This is incongruent with the result of a study conducted in the U.S. [23], in which the decrease rates of the white-collar workers and service workers were larger than those of the blue-collar and farm workers. This may be attributed to the fact that worksites for blue-collar workers have fewer non-smoking designations [27, 28] and blue-collar workers or service workers are more likely to work at places where smoking is allowed [29]. Blue-collar workers have higher smoking rates and intensity with a lower success rate of smoking cessation compared to other worker groups [30, 31]. In Korea, smoking regulations in the workplaces of service and blue-collar workers are not strong enough. Under the KSCO’s major occupational categories, the decrease in cotinine concentration in urine was greatest in managers and smallest in plant and machine operators and assemblers. As the influence of secondhand smoke in occupations varies by worksite smoke-free policies [32], such a result may be attributable to different ratios of smoke-free zone designations and smoking rates by occupation [23, 33].

In this study, the urine cotinine concentration decreased in all subgroups. Such a result is congruent with the result of a study in which serum cotinine levels in various occupation groups all dropped between 1988 and 2002. This shows the impact of smoking regulation policies, including clean indoor air legislation at the state and local level [23, 34, 35].

Smoking restriction policies such as public smoking bans, tax measures, indoor air acts, and mass media promotion have been known to be closely related to smoking rates and subsequent secondhand smoking rates [30]. Similarly, in Korea, the National Health Promotion Act, first enacted in 1995, went through a number of revisions to strengthen measures for smoking cessation. In February 2008, through the revision of Article 9 (Measures for smoking cessation) section 3, the age verification system in cigarette vending machines was strengthened, public facilities were designated as smoke-free zones, separations between smoking areas and non-smoking areas were imposed, and the criteria for ventilation in smoking facilities were toughened through the revision of section 4. In 2010, through the addition of Article 9 section 5, local governments were enabled to designate certain places where many people gathered or travelled within their jurisdiction as non-smoking zones, and to charge a penalty of ≤ 100,000 won (about US$100) in case of violation. Under this policy, local governments are designating places such as bus and taxi stations, parks, playgrounds, tourist sites, crosswalks, streets, and residential areas as non-smoking areas by additional ordinances. Furthermore, through the revision in June 2011, the range of smoke-free public facilities has been extended to the following: restaurants larger than 150 m^2^ in area; sports facilities which can accommodate ≥ 1,000 people including baseball or soccer stadiums; office buildings, factories, and multi-purpose buildings larger than 1,000 m^2^ in area; and private institutions or underground shopping complexes larger than 1,000 m^2^ in area or with ≥ 300 seats.

The results of urinary cotinine concentration were decreased with time in all subgroups in this study. However, decreased level of urine cotinine concentration of the blue-collar and Plant and machine operators and assemblers was lower than the other subgroups. It has been able to determine that these groups are relatively vulnerable to secondhand smoke exposure. Therefore, in these groups, more intervention effort for restriction of smoking in the workplace will be further emphasized.

According to the 2010–2012 National Health Statistics: National Health and Nutrition Examination Survey 4th, 5th < Health and Human Services, Centers for Disease Control>, In Korea adults’ smoking rate after 19 years of male were 46.9 % in 2009, 48.3 % in 2010, 47.3 % in 2011, 43.7 % in 2012 and in female, 7.1 % in 2009, 6.3 % in 2010, 6.8 % in 2011, 7.9 % in 2012 [4, 36, 37]. Comparing the results of this study with the changes in domestic smoking rate, the urine cotinine levels of working groups decreased without obvious reduction of smoking rate. This result may be closely related to the enforcement of the National Health Promotion Act and the subsequent enforcement of worksite smoking policies that have resulted in decreasing of the exposure to secondhand smoke of non-smoking workers in Korea.

We can conclude that the smoking regulations through such strengthened legislation and the subsequent enforcement of worksite smoking policies have influenced non-smoking workers’ exposure to secondhand smoke in Korea and have resulted in the changes in their urine cotinine concentration level.

This study is the first attempt in Korea to indirectly determine the changes over time in workers’ urine cotinine concentration and the effectiveness of the reinforcement of non-smoking regulation policies in Korea.

The limitations of this study include that various sources of environmental exposure to smoke were not controlled. Because of the half-life of cotinine is 18–24 h, cotinine reflects the smoking of 2–3 days, so there will be also related to other outreach sites. By individual, some may be more exposed to smoke in daily activities at home or restaurants than at their workplaces. Particularly, the difference in the exposure to passive smoking at home was not evaluated and controlled. In addition, the composition of the study subjects across years was heterogeneous.

## Conclusions

This study identified the changes in Korean non-smoking workers’ exposure to secondhand smoke through the decrease of the urine cotinine concentration. Such a result may be an indirect indicator of the effectiveness of the revision of the National Health Promotion Act and non-smoking policies in Korea. For future research, a long-term follow-up study on a larger number of subjects should investigate trends in their exposure to ETS and the subsequent changes in their health.
